# First-principles investigation of the structural, optoelectronic, and thermoelectric properties of Sr_2_MgXO_6_ (X = S, Se) double perovskite materials

**DOI:** 10.1038/s41598-026-49506-y

**Published:** 2026-04-25

**Authors:** Ahmad Ali, Aasim Ullah, Mohamed Karouchi, Haroon Khan, Sikander Azam, Hijaz Ahmad

**Affiliations:** 1 Department of Physics, Government Degree College Lahor, Swabi, Pakistan; 2 School of Materials Science and Engineering, Wuhan University of Science and Technology, Wuhan, 430081 P. R. China; 3 Laboratory of Engineering in Chemistry and Physics of Matter, Faculty of Sciences and Technics, Sultan Moulay Slimane University, BP 523, Beni Mellal, 23000 Morocco; 4 Department of Physics, Abdul Wali Khan University, Mardan, 23200 Pakistan; 5 University of West Bohemia, New Technologies – Research Centre, 8 Univerzitní, Pilsen, 306 14 Czech Republic; 6 Faculty of Engineering and Applied Sciences, Department of Physics, Riphah International University, Islamabad, Pakistan; 7 Department of Mathematics, Faculty of Science, Islamic University of Madinah, Madinah, 42351 Saudi Arabia; 8 Irfan Suat Gunsel Operational Research Institute, Near East University, Nicosia/TRNC, 99138 Mersin 10 Turkey; 9 VIZJA University, Okopowa 59, 01-043 Warsaw, Poland; 10 Department of Mathematics, College of Science, Korea University, 145 Anam-ro, Seongbuk-gu, Seoul, 02841 South Korea; 11 Engineered Biomaterials Research Center, Khazar University, Baku, Azerbaijan

**Keywords:** DFT Calculations, Chalcogenides, Double Oxides Perovskites, Structural Properties, Optoelectronic properties, Energy science and technology, Materials science, Optics and photonics, Physics

## Abstract

The physical properties of the chalcogenide-based double perovskites Sr₂MgXO₆ (X = S, Se) were investigated in the cubic phase using first-principles density functional theory. Structural stability was assessed through formation energy analysis and ab initio molecular dynamics simulations, confirming both dynamical and thermal stability of the compounds. The electronic structure was calculated using the full-potential linearized augmented plane wave method in combination with the Tran–Blaha modified Becke–Johnson potential, yielding improved band-gap values of 1.4 eV for Sr₂MgSO₆ and 2.2 eV for Sr₂MgSeO₆. Real-space bonding characteristics were examined using electron localization function, charge density difference, and Bader charge analyses, revealing dominant ionic interactions with moderate covalent contributions within the chalcogen–oxygen framework. Carrier transport properties were evaluated through electron and hole effective mass calculations, indicating relatively favorable electron mobility in both materials. Using the effective masses and static dielectric constants, exciton binding energies were estimated and found to be sufficiently low to enable efficient exciton dissociation at room temperature. Optical properties, including dielectric response, absorption coefficient, reflectivity, energy loss function, and refractive index, demonstrate strong absorption across the visible to ultraviolet regions with low optical losses. The combined electronic, bonding, excitonic, and optical characteristics highlight Sr₂MgXO₆ (X = S, Se) as promising candidates for photovoltaic and optoelectronic applications. Thermolectric study suggests that the materials are p-type semiconducting due to their positive Seebeck values. The PF and ZT analyses indicate that Sr₂MgSeO₆ exhibits higher thermoelectric performance compared to Sr₂MgSO₆ material.

## Introduction

The growing demand for sustainable clean energy solutions arises from the extensive and significant impacts of existing environmental challenges, including ecological disruption, public health risks, and overarching threats to global biodiversity. It is anticipated that nonrenewable energy sources such as coal and gas will run out by the end of 2050^1^. Researchers are exploring novel approaches to developing technology for generating energy from renewable sources to address the modern era’s growing energy demand and scarcity^2,3^. Recent in-depth studies on CH_3_NH_3_PbX_3_ (X = Cl, Br, I), an organic-inorganic hybrid halide perovskite (OIHP), have completely changed the solar cell industry. The lab-scale record power conversion efficiency (PCE) has been revised from 3.8% in 2009 to 34% in 2025^4,5^.

Perovskite materials represent a significant technological advancement, characterised by a distinctive crystalline structure that enables exceptional adaptability across a range of essential applications. Double perovskites (DPs) possess unique features that have attracted greater attention due to their potential for renewable energy applications^6,7^. The double perovskites are widely found as hydroxides, sulfides, nitrides, and halides^8^. However, when exposed to light, moisture, oxygen, and ultraviolet radiation, perovskite degrades due to its toxicity and instability^9^. The capacity of perovskite materials to absorb visible radiation should also be considered when assessing their suitability for solar cells^10^. Researchers have been actively developing Pb-free PSCs to examine potential large-scale solar applications, while DPs have emerged as a promising alternative. DPS, generally characterized by the formula A_2_BCX_6_ (where A, B, and C are metal cations and X is an anionic element, halide, or Oxide), has formed the basis for the development of several sectors, including photovoltaic, optoelectronic, photocatalytic, and thermoelectric energy^11–13^. All of the elements in group sixteen of the periodic table, specifically Sulphur (S), Selenium (Se), and Tellurium (Te), are referred to as chalcogens, and compounds that contain at least one chalcogen are called chalcogenides^14^. These materials exhibit exceptional properties, including strong thermal and aqueous stability, and optoelectronic characteristics^15^. As a result, chalcogenides have attracted the attention of numerous researchers as a potential material for metal oxide composites. Chalcogenide perovskites were recently proposed as potential solar cell absorbers to overcome the inherent instability of halide perovskites. This is because the Coulomb interaction in chalcogenides is anticipated to be four times greater than that of halides for purely ionic systems^4^. The general formula for chalcogenide-based perovskite materials is ABX_3_, where X stands for a chalcogen element, A for a bigger cation, and B for a smaller cation with a + 4 oxidation state^16^.

Sun et al.^17^ reported and theoretically investigated chalcogenide-based perovskites of the form ABX_3_ (where A stands for Ca, Sr, or Ba, B for Ti, Zr, or Hf, and X for S or Se) as solar energy absorbers. Assoud et al.^18^ have synthesised Sr_2_SnSe_5_ and SrSn_2_Se_4_ materials; the predicted energy band gaps are 0.9 eV and 0.2 eV, respectively. The remarkable optical characteristics and kinetic and thermodynamic stability of Ca_2_GaNbS_6_, Ba_2_AlNbS_6_, Ba_2_SnHfS_6_, and Sr_2_InNbS_6_ were reported and investigated theoretically by Agiorgousis et al.^19^. Bendjilali et al.^20^ conducted first-principles simulations to determine the structural, elastic, electrical, optical, and thermoelectric (TE) properties of Z_2_GaNbS_6_ (where A = Ca, Sr, and Ba). They proposed chalcogenides based double perovskites as possible choices for TE applications. Kassa et al.^21^ have carried out a first principles study of Ca_2_ZrHfS_6_ material, the material is found to be semiconducting with band gap of 0.95 eV, and reported to be potential candidate for solar cells. Adjogri et al.^22^ explored double chalcogenide perovskites to identify novel photovoltaic absorbers that can replace lead based hybrid halide perovkites. A Ali et al.^23^ have reported the double perovskite materials family Ba_2_UXO_6_ (X = Co, Mn), for optoelectronic and magnetic applications, using a DFT study. The materials are found to be half-metallic and ferromagnetic. A Ali et al.^24^ have also made first-principles investigations of Ba_2_GdXO_6_ (X = Nb, & U) materials by computing the magnetic and optoelectronic characteristics. The materials are found to be ferromagnetic and semiconducting. Malak Azmat et al.^25^ have reported Ba_2_MgXO_6_ (X = Se, S) materials and investigated there physical properties by utilizing DFT study. The materials exhibit narrow energy band gaps. The first principles investigations of Ba_2_CaXO_6_ (X = Se, S) are carried out to study their optoelectronic, thermoelectric, and mechanical properties by Albalawi.^26^.

The literature review mentioned above suggests that chalcogenide-based double perovskites can be utilized in solar cells and thermoelectric power generators. The materials under study are chosen based on investigations by Ullah et al.^27^, who have focused on the structural, optoelectronic, and mechanical studies of the Sr_2_MgXO_6_ (X = Se, S) compounds, during their investigations. Unlike previous reports on chalcogenide and oxide double perovskites that primarily focus on electronic and optical characteristics, the present study establishes a comprehensive structure–property framework by jointly analyzing bonding, carrier effective masses, and exciton binding energies for the previously unexplored Sr₂MgXO₆ (X = S, Se) compounds. Furthermore, thermoelectric properties of the materials have also been investigated in this study, which have not been explored previously. The results shed light on the fundamental properties of these materials and provide new insights for their practical applications.

## Computational method

The computations for this study were carried out using Wien2k Software, which is based on density functional theory (DFT)^28^. The full-potential linearized augmented plane wave (FP-LAPW) approach incorporated in Wein2k to solve the Kohn–Sham equation for electron-ion interaction^29^. To solve the Kohn–Sham equation’s exchange-correlation energy (Exc), the Tran–Blaha modified Becke–Johnson potential (TB-mBJ) has been employed to obtain accurate energy band gaps and related properties, including structural, optical and thermoelectric properties of the materials^28^. The TB-mBJ potential was selected due to its demonstrated ability to reproduce semiconductor band gaps with substantially improved accuracy over standard GGA while maintaining semilocal computational cost. For prototypical semiconductors such as Si, Ge, GaAs, and SiC, TB-mBJ typically reproduces experimental band gaps within ~ 0.1–0.3 eV. Broader benchmark studies across chemically diverse material sets report mean absolute errors of ~ 0.4–0.6 eV, confirming competitive performance for a semilocal approach^30–32^. Hybrid functionals such as HSE06 yield comparable overall accuracy but at significantly higher computational expense, while GW methods can further improve quasiparticle gap predictions (~ 0.1–0.2 eV accuracy) at substantially greater computational cost. TB-mBJ, therefore, represents a deliberate accuracy and efficiency compromise appropriate for the present study. A quantitative comparison of TB-mBJ, HSE06, and GW band gaps for representative semiconductors is provided in Table 1 of the Supporting Information. To ensure the validity of our findings, systematic k-point convergence tests were conducted over a variety of values, such as 100, 300, 500, 1000, and 2000. This enabled us to establish the minimum density required to achieve complete convergence of the energy within our desired precision, while keeping the computational effort viable. The optimized k-point value chosen is 1000 (10 × 10 × 10) in the Brillouin zone. The optimized k-point value is further used in optical and DOS calculations. A cut-off energy of -6 Ry was chosen for the computations. To prevent the current leakage from the core, the muffin-tin radii (RMT) values of the material’s elements, for Sr_2_MgSO_6_, are 2.1 (Sr), 1.90 (Mg), 1.5 (S) and 1.2 (O) and for Sr_2_MgSeO_6_, are 2.1 (Sr), 1.90 (Mg), 1.75 (Se) and 1.60 (O). In the interstitial zone between the muffin tin spheres, plane wave expansions were used, while spherical harmonics were used to expand the wave electron charge densities and potentials inside the spheres. The charge density was stretched to G_max_ = 11 and a cut-off value of I_max_ = 9. To obtain accurate computational results *RMT* × *K*_max_= 7 has been chosen. Here, *K*_max_ refers to the maximum reciprocal lattice vector employed in the expansion of plane waves, whereas RMT denotes the minimum radius of the smallest muffin-tin (MT) sphere. The self-consistent computations are said to have converged when the crystal’s total energy reaches 10^− 4^ Ry and charge to 10^− 3^ e. To determine the optical characteristics, the complex dielectric function of the material has been calculated, which is:

1 $$\:\epsilon\left(\omega\:\right)={\epsilon\:}_{1}\left(\omega\:\right)+{\epsilon\:}_{2}\left(\omega\:\right)$$

The Kramer’s-Kronig relations were used to get the real component ε_1_ and the imaginary part ε_2_. Further, both ε_1_ and ε_2_ can be expressed mathematically by the following two Eq. 

2 $$\:{\epsilon\:}_{1}\left(\omega\:\right)=1+\:\frac{2}{\pi\:}P{\int\:}_{0}^{\infty\:}\frac{\omega\:{\epsilon\:}_{2}\left(\omega\:\right)}{{\omega\:`}^{2}-{\omega\:}^{2}}\:d\omega\:$$

3 $$\:{\epsilon\:}_{2\left(\omega\:\right)\:=\:}\frac{{e}^{2}h`}{\pi\:{m}^{2}{\omega\:}^{2}}{\sum\:}_{\begin{array}{c}v,c\\\:\:\end{array}}\:\int\:BZ{\left|{M}_{cv}\left(K\right)\right|}^{2}\delta\:\left[{\omega\:}_{cv}\left(K\right)-\omega\:\right]{d}^{3}kz$$

The real and imaginary parts of the dielectric function are used to characterizes the material’s optical nature. Furthermore, the absorption coeffiecent, refractive index, reflectivity and energy loss functions, extinction coefficient and optical conductivity of the materials are computed using the following mathematical relations^33–35^;

4 $$\:\mathrm{I}\left(\omega\:\right)=\:\frac{\sqrt{2}\omega\:}{c}{\:\:\left(\:\sqrt{{\epsilon\:}_{1}{\left(\omega\:\right)}^{2}+{\epsilon\:}_{2}{\left(\omega\:\right)}^{2}}-{\epsilon\:}_{1}\left(\omega\:\right)\right)}^{\frac{1}{2}}$$

5 $$\:n\left(\omega\:\right)=\:{\left[\frac{1}{2}\:\left(\genfrac{}{}{0pt}{}{\:}{\:}\sqrt{{\epsilon\:}_{1}{\left(\omega\:\right)}^{2}+{\epsilon\:}_{2}{\left(\omega\:\right)}^{2}}\:+\:{\epsilon\:}_{1}\left(\omega\:\right)\right)\:\right]}^{1/2}$$

6 $$\:R\left(\omega\:\right)=\:{\:}^{\:}{\left|\frac{\sqrt{{{\epsilon\:}_{1}}^{\:}\left(\omega\:\right)\:+\:\mathrm{i}{\epsilon\:\left(\omega\:\right)}^{\:}}\:-1\:\:\:}{\sqrt{{\epsilon\:}_{1}\left(\omega\:\right)\:+\mathrm{i}{\epsilon\:\left(\omega\:\right)}_{\:}}\:\:\:\:+1\:\:\:\:}\right|}^{2}$$

7 $$\:L\left(\omega\:\right)=\:\:\frac{{\epsilon\:}_{2\:\:}\left(\omega\:\right)}{{\epsilon\:}_{1}{\left(\omega\:\right)}^{2}+\:{\epsilon\:}_{2}{\left(\omega\:\right)}^{2}\:}$$

8 $$\:\sigma\:\left(\omega\:\right)={\sigma\:}_{1}\left(\omega\:\right)+i\:{\sigma\:}_{2}\left(\omega\:\right)=i\:\frac{\omega\:}{4\pi\:}\left[\epsilon\:\left(\omega\:\right)-1\right]$$

9 $$\:\kappa\:\left(\omega\:\right)={\left[\frac{\sqrt{{\epsilon\:}_{1}{\left(\omega\:\right)}^{2}+{\epsilon\:}_{2}{\left(\omega\:\right)}^{2}}}{2}-\frac{{\epsilon\:}_{1\:\:}\left(\omega\:\right)}{2}\right]}^{\raisebox{1ex}{$1$}\!\left/\:\!\raisebox{-1ex}{$2$}\right.}$$

## Results and discussions

The results obtained from the investigations of chalcogenide-based double perovskite oxide Sr_2_MgXO_6_ (X = S, Se) are discussed in the following sections.

### Structural Properties

**Fig. 1:**
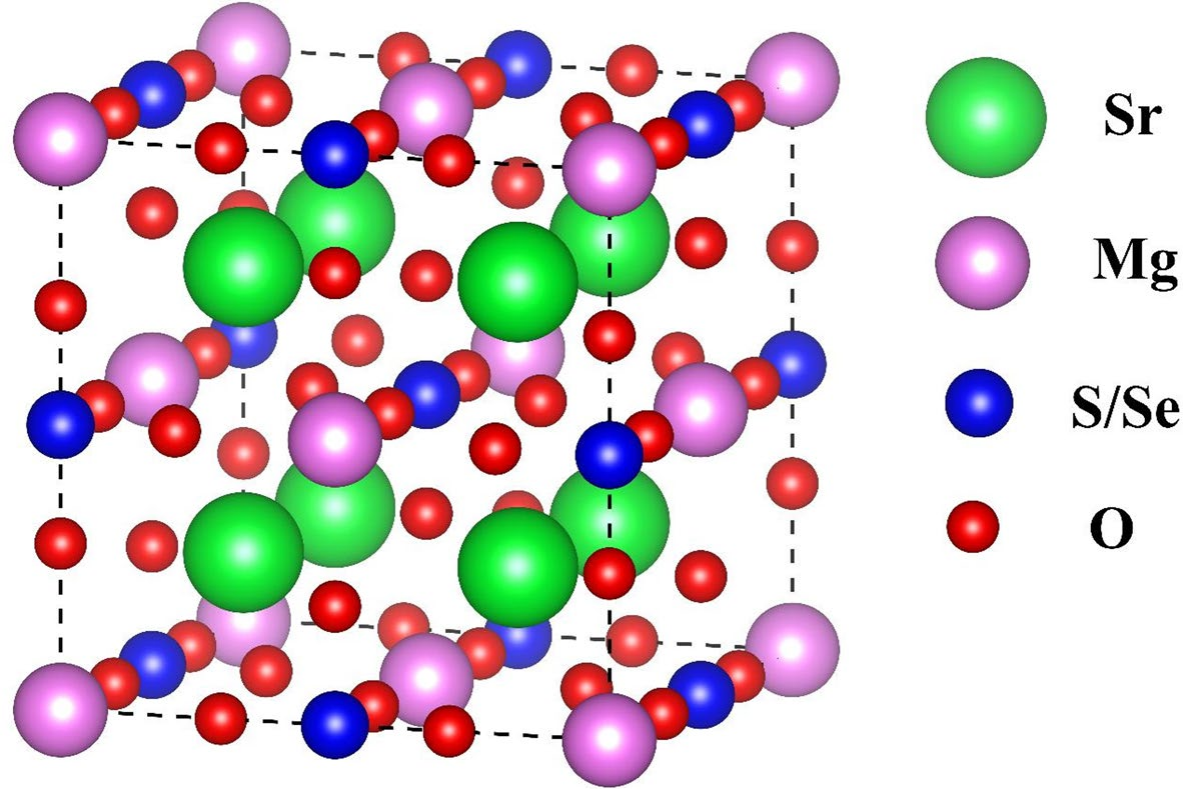
The Crystallographic Structure of Sr_2_MgXO_6_ (X = S, Se) Double chalcogenides perovskites.

The Sr_2_MgXO_6_ (X = S, Se) materials are crystalized in a cubic (Fm-3 m) space group, and the crystallographic structure is displayed in Fig. 1. The stability of crystallographic phases in double perovskite materials can be assessed through the prediction of the tolerance factor τ associated with these materials. In a variety of crystal structures, such as perovskites, garnets, and pyrochlores, phase stability is predicted by the tolerance factor, a structural descriptor^36^. Forecasting the stability of the perovskite structure remains a persistent challenge in the quest for new functional materials applicable in various fields such as photovoltaics and electro-catalysts^37^. The tolerance factors (τ) of the materials have been computed using the Goldschmidt tolerance factor^38^, as given by the relation,

10 $$\:\tau\:=\frac{{r}_{Sr}+{r}_{O}}{\sqrt{2}\left(\frac{{r}_{Mg}+{r}_{(X=Se\:\&\:S)}}{2}+\:{r}_{O}\right)}$$

The r_Ba_, r_Ca_, r _(M=Se, S)_, and r_O_ represent the ionic radii of the constituent elements of the materials. The respective values of these radii are 1.44 Ȧ (r_Sr_), 0.72 Ȧ (r_Mg_), 0.19 Ȧ (r_Se_), 0.12 Ȧ (r_S_), and 1.40 Ȧ (r_O_). The ionic radii chosen for computing the tolerance factor of the materials are sourced from Shannon (1976)^39^. Based on these data, the predicted values of the tolerance factors for Sr_2_MgSeO_6_ and Sr_2_MgSO_6_ are 1.067 and 1.086, respectively. The tolerance factor value for the stable cubic structure is 1. The tolerance factor of the materials predicts slight distortion from (τ = 1) the stable cubic phase.

One of the most crucial characteristics of a compound that has a direct bearing on stability is formation energy. Machine learning techniques have proven effective for predicting the formation energy and thermodynamic stability of materials, which are essential for materials discovery. These models employ extensive datasets derived from density functional theory (DFT) calculations to efficiently predict formation energies, minimizing the need for substantial computational resources. A compound is more likely to be stable if its formation energy is negative^40^. Thermodynamic stability of the materials can be predicted by computing the formation energies of the materials under study using the relation,

11 $$\:\varDelta\:{H}_{F}=\:\frac{{E}_{T\left({Sr}_{2}MgX{O}_{6}\:\left(X\:=\:Se\:\&\:S\right)\right)}-\left(2{E}_{Sr}+\:{E}_{Mg}+\:{E}_{\:X\:=\:Se\:\&\:S}+\:6{E}_{O}\right)}{10}$$

**Fig. 2:**
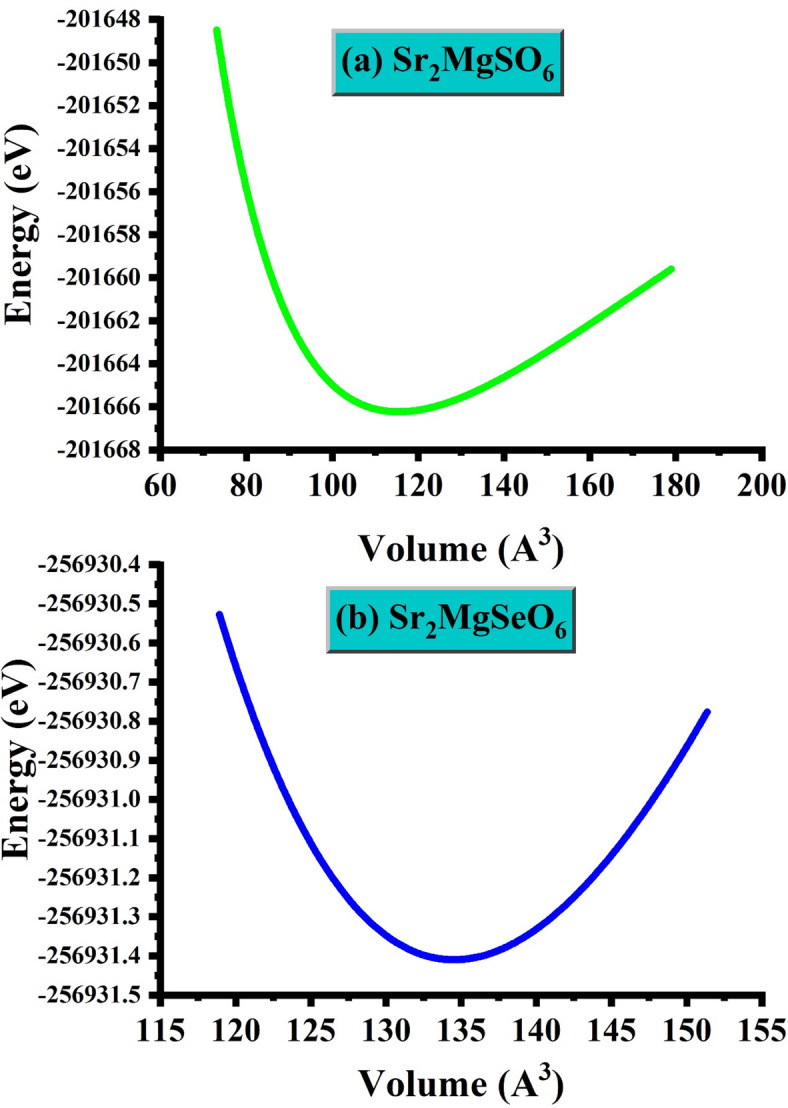
The energy vs. volume optimization curves of (**a**) Sr_2_MgSO_6_ and (**b**) Sr_2_MgSeO_6_ materials.

The expression of formation energy used is the thermodynamic definition of enthalpy of formation and is defended in DFT by the approximation of the enthalpy by the total ground-state energy at zero temperature and pressure. The equation quantifies of the energy difference between the compound and its constituent parts in their most stable reference states, which is a criterion of thermodynamic stability. The E_T_, E_Sr_, E_Mg_, E_X_ and E_O_ are energies of the compounds, Sr, Mg, X = Se, S and O elements, respectively. The energies are computed by performing energy vs. optimizations, displayed in Fig. 2 (a & b), of both materials. The predicted formation energies per atom of the Sr_2_MgSO_6_ and Sr_2_MgSeO_6_ materials are − 3.49 eV and − 3.36 eV, respectively. The negative formation of the materials suggests that their synthesis is thermodynamically feasible. Table 2 presents the overall structural parameters, including optimized atomic positions, lattice constants, volume, bulk modulus, formation energy, and tolerance factors of the materials under study.

**Fig. 3:**
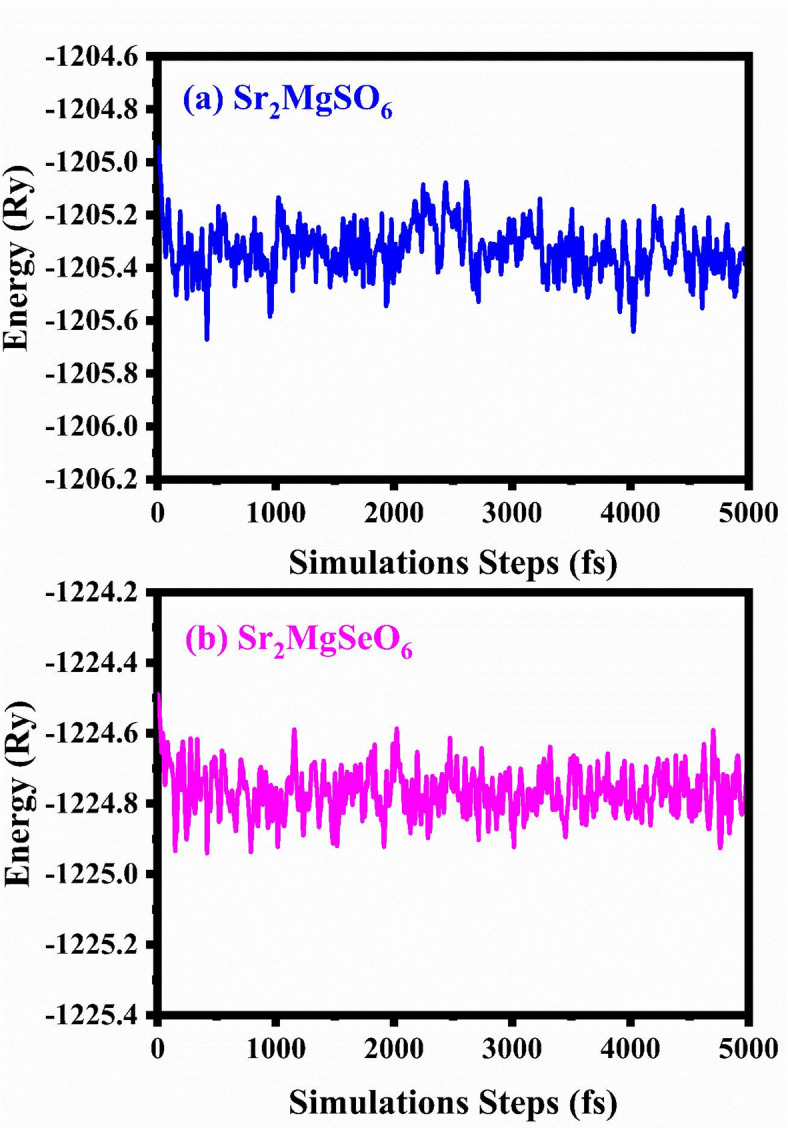
The AIMD simulations of (**a**) Sr_2_MgSO_6_ and (**b**) Sr_2_MgSeO_6_ materials.

The ab initio molecular dynamics (AIMD) simulations of the materials have been carried out to check the thermodynamic stability of the materials by the DFT-based Quantum Expresso code. The AIMD simulation plots are shown in Fig. 3 (a & b). The stability analysis indicates that both materials exhibit stable total energy profiles with little change, indicating structural integrity at the simulated temperature. The Sr₂MgSO₆ demonstrates a peak energy variation of roughly 0.6 Ry, whereas Sr₂MgSeO₆ presents a marginally reduced fluctuation of approximately 0.4 Ry, indicating superior thermal stability. The increased stability of Sr₂MgSeO₆ may be ascribed to the effect of selenium, which likely fosters more robust bonding connections inside the lattice. The thermal resilience of these materials underscores their suitability for high-temperature applications in electrical and energy devices.

**Table 1:** Benchmark comparison of TB-mBJ, HSE06, and GW for representative semiconductors.

Material	Experimental Gap (eV)	TB-mBJ (eV)	HSE06 (eV)	GW (eV)	Typical Deviation (TB-mBJ)
Si	1.17	~ 1.17	~ 1.20	~ 1.25	~ 0.0–0.1 eV
Ge	0.74	~ 0.85	~ 0.75–0.80	~ 0.90	~ 0.1 eV
GaAs	1.52	~ 1.60	~ 1.40–1.55	~ 1.60	~ 0.1–0.2 eV
SiC	2.40	~ 2.30	~ 2.20–2.40	~ 2.40–2.50	~ 0.1–0.2 eV

**Table 2:** The structural parameters, including optimized atomic positions, lattice constants, volume, bulk modulus, formation energy, and tolerance factors, for Sr_2_MgXO_6_ (X = S, Se) double chalcogenide oxide materials.

Materials	Atoms	x	y	z	a (A)		B (GPa)		Vo (A^3^)		E_0_ (Ry)	∆H_f_ (eV/atom)		τ
**Sr**_**2**_ **MgSO**_**6**_	Sr	0.25	0.25	0.25	Present Study	Previous Study^27^	Present Study	Previous Study^27^				Present Study	Previous Study^27^	
	Mg	0.00	0.00	0.00										
	S	0.00	0.00	0.50	7.73	7.62	136.16	127.43	462.46		-14822.23	−3.49	-4.61	1.08
	O	0.00	0.00	0.30										
**Sr**_**2**_ **MgSeO**_**6**_	Sr	0.25	0.25	0.25										
	Mg	0.00	0.00	0.00										
	Se	0.50	0.00	0.00	8.13	7.77	121.94	129.27	981.48		-18884.11	−3.36	-4.66	1.06
	O	0.00	0.00	0. 29			`							

### Electronic properties

**Fig. 4:**
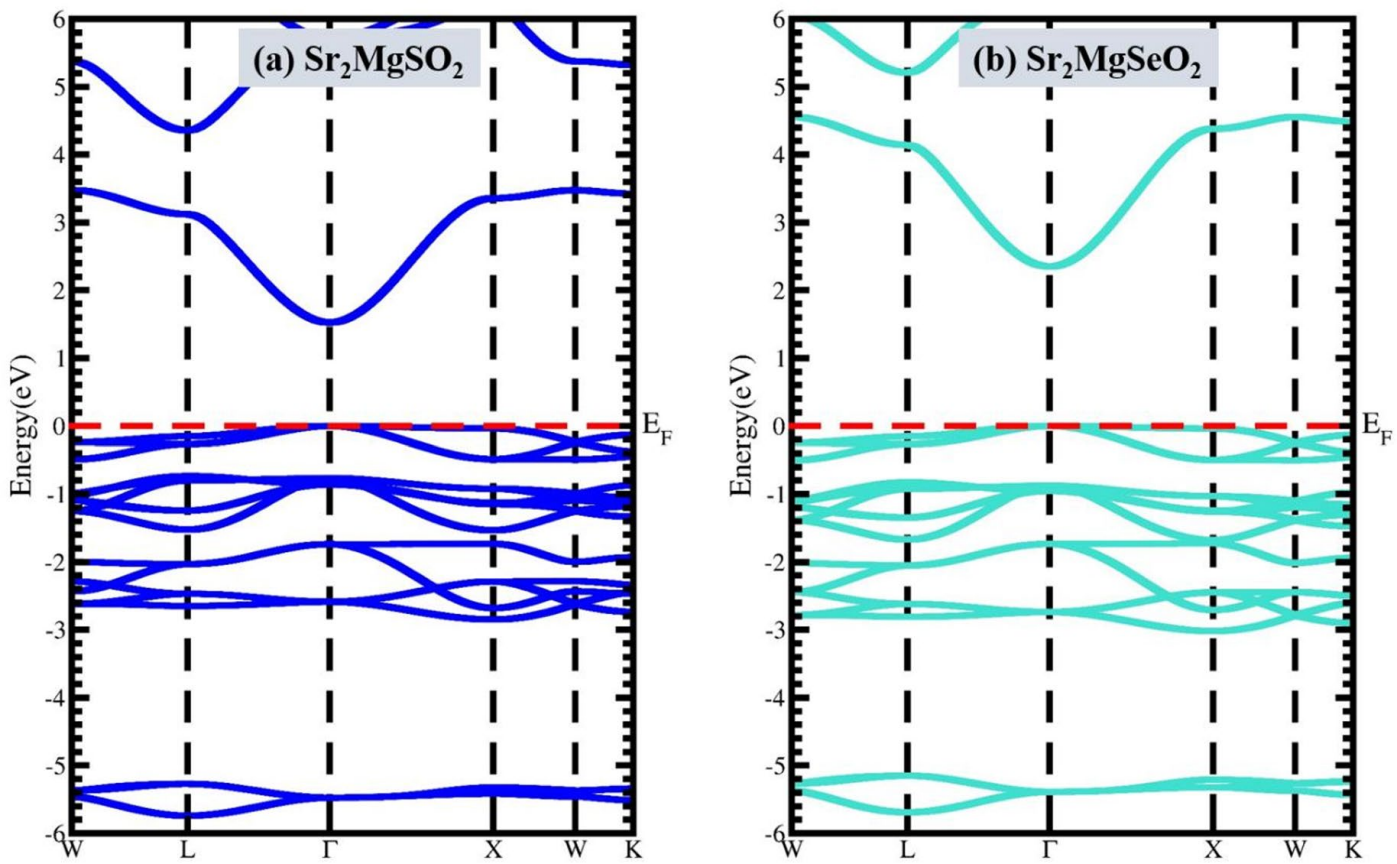
The electronic band structures of (**a**) Sr_2_MgSO_6_ and (**b**) Sr_2_MgSeO_6_ materials.

**Fig. 5:**
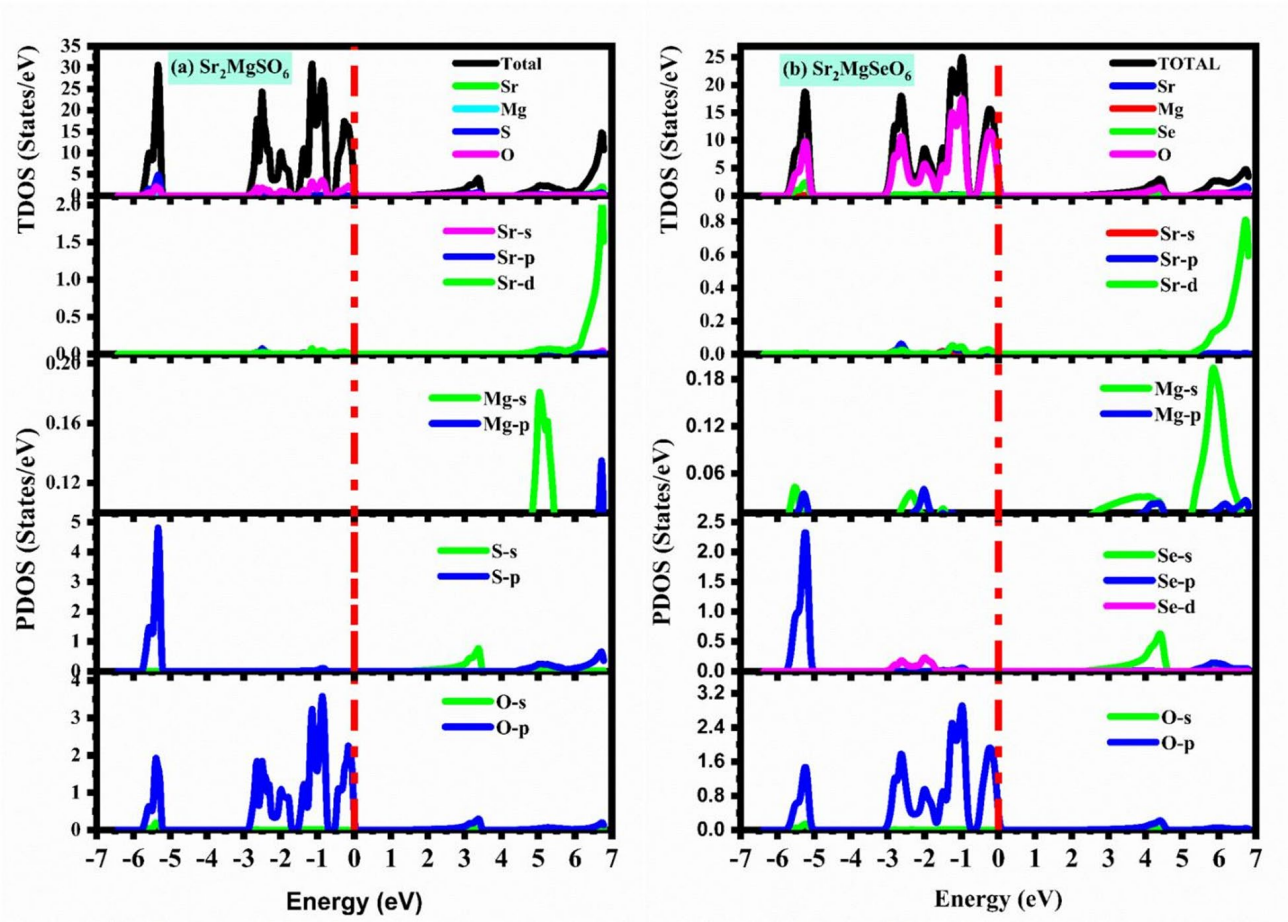
The Total (TDOS) and Partial (PDOS) of the (**a**) Sr_2_MgSO_6_ (**b**) Sr_2_MgSeO_6_ materials.

Comprehending and adjusting the electronic properties of double perovskites for diverse applications requires an in-depth grasp of electronic band structures and density of states. The material’s electrical properties can be manipulated by engineering its band structure. To optimize charge-carrier transit, which impacts the performance of electrical devices such as solar cells, transistors, and sensors, a well-defined density of states is crucial^41^. The electronic properties in terms of band structure (BS) and density of states (DOS) are computed and shown in Figs. 4 and 5, respectively, to examine the suitability of the studied chalcogenides-based double perovskite oxide for various applications. The TB-mBj approximations have been used to determine electronic properties, as numerous studies have demonstrated that TB-mBj^42^ can accurately calculate band gaps. The electronic properties of materials are determined by the location of the Fermi level and the energy band gaps between the valence band maximum (VBM) and conduction band minimum (CBM). The electronic band structure has been calculated to identify potential transitions from the Valence band (VB) to the Conduction band (CB)^43^. The electronic band structures of Sr_2_MgXO_6_ (X = S, Se) has been displayed in Fig. 4 (a & b), which demonstrates that Sr_2_MgSO_6_ have direct band gap semiconducting nature of the material, as the conduction band maxima (CBM) lie at the “Γ” and valence band minima (VBM) yield to an axis at “L” with a tiny gap of 1.4 eV energy. The direct band gap semiconductor has wide applications, including photovoltaic applications^44^, and photonic devices^45^. This indirect band gap semiconductor has recently become favorable for high-power and high-temperature^46^. The Sr_2_MgSeO_6_ has a direct band gap because the CBM and VBM both lie at the “Γ” point with a narrow gap of 2.2 eV, making it a direct band gap semiconductor. Thus, both the materials under study is narrow band gap semiconductors. Materials with a direct band gap can be used in LEDs because they can undergo electronic state transitions without altering momentum, which is crucial for photon absorption^47^. The density of states describes the contribution of each energy level or quantum state to the overall properties of a material, such as its electronic, thermal, optical, and magnetic behavior. To visualize the electrical origin of the band structure, examine the bonding properties of our materials, and determine the contributions of various electron states, the density of states of the materials has been calculated. The density of states provides insight into the electronic nature of the materials. The elemental contribution is explained by the total density of states (TDOS), and the electronic states contribution is given by the partial density of states (PDOS) of the materials. The DOS results of both materials are depicted in Fig. 5 (a & b). The valence band for Sr_2_MgSO_6_ is from 0 to -6 eV, and for Sr_2_MgSeO_6_ is from 0 to -5.8 eV of energy. The PDOS of both materials indicates the hybridization of Sr-d and O-p states in the energy range of 0 to -3 eV for Sr_2_MgSO_6_ and 0 to -2.2 eV for Sr_2_MgSeO_6_ material. The DOS confirms the energy band gaps of both materials under study. The TDOS plots suggest that for both materials, the “O” provides major contributions near the Fermi level, and other elements have smaller or negligible contributions to the electronic conductivity. The PDOS of both materials indicates that the valence band maxima are formed by O-p and Sr-d states, and the conduction band minima are formed by O-p states. The band gaps indicate that the materials are good options for photovoltaic and solar cell applications^48^, which have wide applications like photovoltaic^49^, nanorods^50^, solar cells^51^, optical devices^52^, and detectors^53^.

### Optical properties

**Fig. 6:**
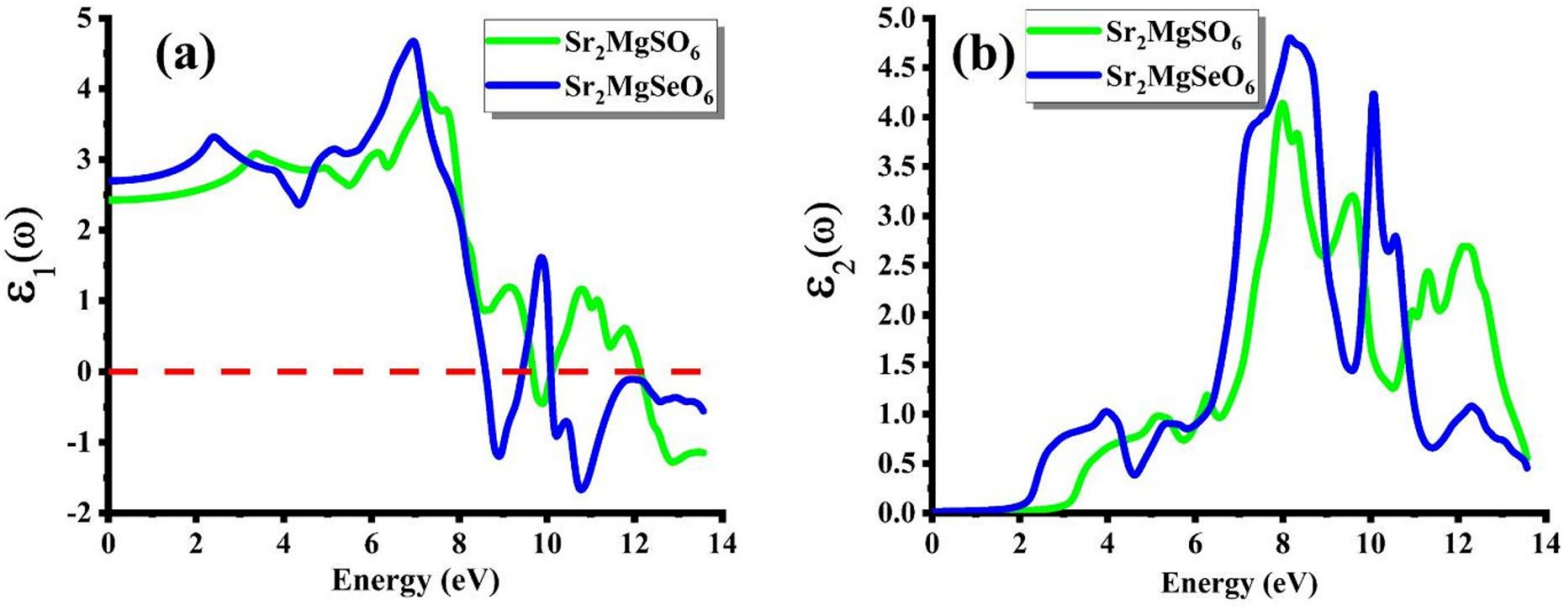
The optical parameters; (**a**) real part; ε_1_(ω), and (**b**) imaginary part; ε_2_(ω) of the Sr₂MgXO₆ (X = S, Se) materials.

**Fig. 7:**
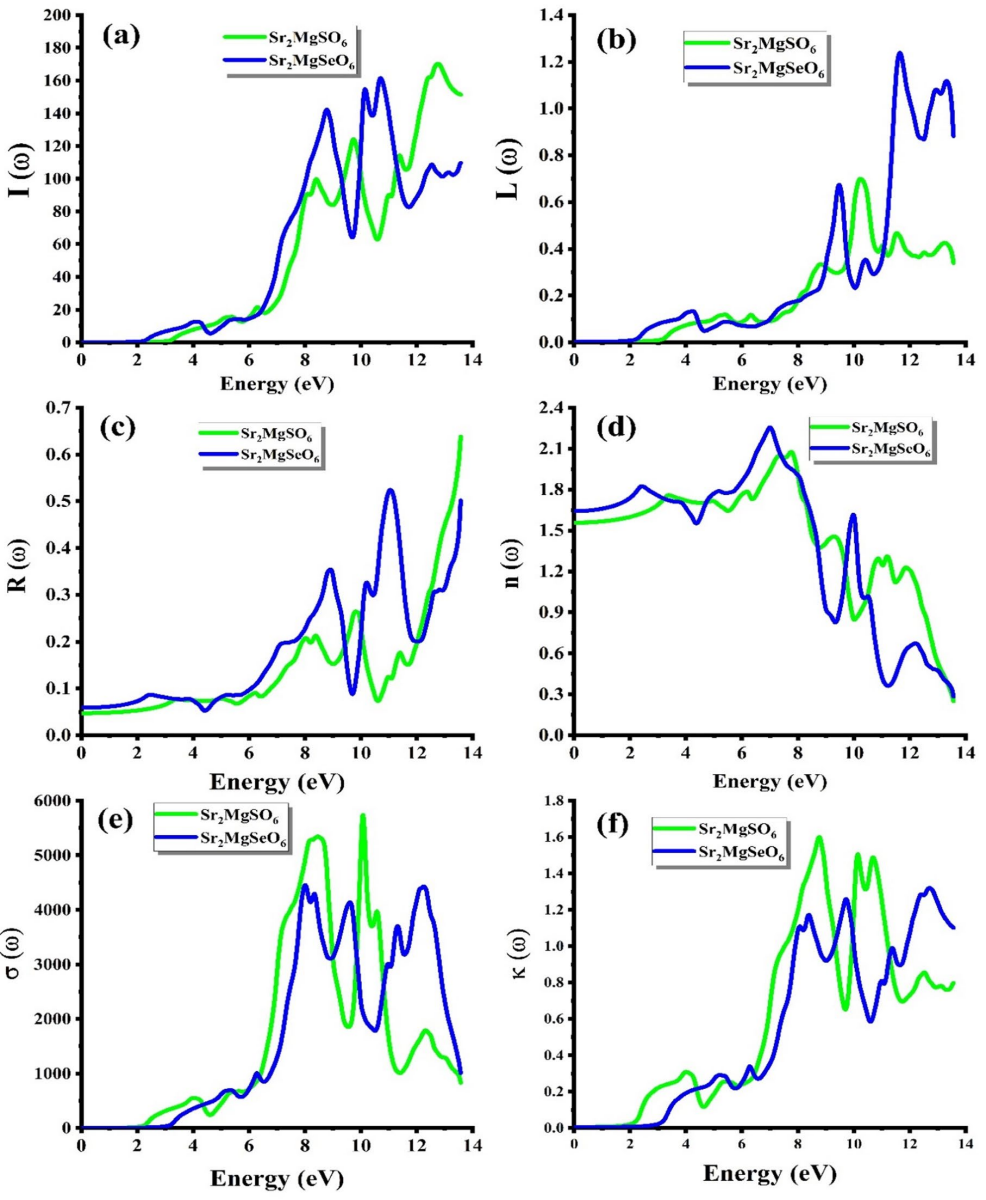
The optical parameters of Sr₂MgXO₆ (X = S, Se); (**a**) absorption coefficient; I (ω), (**b**) energy loss function; L (ω), (**c**) reflectivity coefficient; R (ω), (**d**) refractive index n (ω), (**e**) optical conductivity σ (ω) and (**f**) extinction coefficient κ (ω).

The optical spectroscopy assessment serves as an effective method for assessing the overall band behavior of a solid^54^. The optical properties are crucial for optoelectronic materials; therefore, the optical parameters of the materials have been computed to study their applications in optoelectronics applications, including solar cells. The Figs. 6 and 7 displays the computed optical parameters of Sr_2_MgXO_6_ (X = S, Se) materials. The electronic complex version of the dielectric function ε(ω) offers a comprehensive insight into the optical behavior of solid materials. The computed real and imaginary parts are displayed in Fig. 6 (a & b). The imaginary component of the dielectric function, ε_2_(ω), indicates the material’s absorption or energy loss, which is associated with the electronic band structures. Meanwhile, the energy stored is represented by the real part, ε_1_(ω)^55^. The static dielectric constant or real part ε_1_(0) displayed in Fig. 6 (a) is the value of a compound’s dielectric constant at zero frequency. The static real component ε_1_(0) for Sr_2_MgSeO_6_ and Sr_2_MgSO_6_ have the values of 2.4 and 2.7, respectively. The higher value of Sr_2_MgSO_6_ indicates strong covalent bonding compare to Sr_2_MgSeO_6_ material. The Penn’s model, which states that the static real part ε_1_(0) of a material is proportional inversely to its energy band gaps, can be seen to be valid in these materials. The band gap of Sr_2_MgSeO_6_ is larger than Sr_2_MgSO_6_’s band gap, while the ε_1_(0) of Sr_2_MgSeO_6_ is smaller (2.4) than that of Sr_2_MgSO_6_ (2.7). The real component ε_1_(ω) of the substances under investigation began to decline after reaching its peak value and ultimately fell to a particular negative value within a given frequency range. The real parts ε_1_(ω) of Sr_2_MgSO_6_ and Sr_2_MgSeO_6_ have maximum values of 4.2 and 4.7 at energies of 7.8 eV and 8.2 eV, respectively. The varying peak values and positions suggest unique electronic structures and bonding properties of the two materials. Figure 6 (b) shows ε_2_(ω), the imaginary part of the function for the materials. The plots indicate that ε_2_(ω) for each material has a threshold value each material. The threshold values correspond to the material’s energy band gaps; when incident radiation exceeds these values, the absorption begins. The maximal values of ε_2_(ω) for Sr_2_MgSeO_6_ and Sr_2_MgSO_6_ at 1 eV and 5.5 eV, respectively. Additionally, Sr_2_MgSeO_6_ peaks are located at energies of 1.8 eV and 8.8 eV, whereas Sr_2_MgSO_6_ peaks are located at 3.2 eV and 7.9 eV energies in the optical spectra. Since the energy bandgap determines the absorption threshold, perovskites with smaller band gaps exhibit greater absorption in the visible spectrum^56^. The absorption coefficient can be computed using the imaginary and real components of the dielectric function. The band gap and molecular structure of the material affect the absorption values. As the frequency of an incoming photon resonates with the transition frequency of the atom, optical absorption occurs. The frequency dependence of the absorption coefficient means that only certain materials can absorb photons at certain frequency. The transition of electrons starts from occupied states in the higher valence band to accessible unoccupied levels in the lower conduction band, which is the process that causes optical absorption^57^. The absorptive nature of the materials can be understood by studying the absorption coefficient, displayed in Fig. 7 (a), of Sr_2_MgSeO_6_ and Sr_2_MgSO_6_ materials. The maximum absorption of the light photons for Sr_2_MgSeO_6_ and Sr_2_MgSO_6_ takes place at 10.2 eV and 12.5 eV, respectively. Additional absorption peaks can be found in the absorption spectra for Sr_2_MgSeO_6_ at energies of 1.8 eV, 6.2 eV, 10.5 eV, and 12.8 eV, and for Sr_2_MgSO_6_ at energies of 1.2 eV, 8.6 eV, and 11.3 eV. The materials’ absorption spectra indicate that absorption occurs between the visible and ultraviolet regions of the spectra. Based on the energy band gaps and absorption peaks in the visible regions, the materials appear to be good candidates for solar energy applications. The materials Sr_2_MgSeO_6_ and Sr_2_MgSO_6_ have an energy loss function, as shown in Fig. 7 (b), are minimal for both materials up to 5 eV and slightly increases beyond the mentioned energy range. The energy loss function for Sr_2_MgSO_6_ and Sr_2_MgSeO_6_ is maximum at 10 eV and 11.58 eV, respectively. The energy loss of all materials in the visible region is negligible when compared to UV radiation. The reflectivity of any given material can be used to explain how its surface responds to the incident light photons. The smoothness or roughness of the materials under study is predicted by their reflectivity coefficient; materials with higher reflectivity values are assumed to have smooth surfaces and exhibit lower light photon absorption, while materials with lower reflectivity have rough surfaces and higher incident photon absorption^58^. Figure 7 (c) displays the reflectivity of the materials R (ω) of both materials under study. The static reflectivity of Sr_2_MgSO_6_ and Sr_2_MgSeO_6_ is 0.04 (4%) and 0.06 (6%), respectively. The materials Sr_2_MgSeO_6_ and Sr_2_MgSO_6_ have maximum reflectivity at 13.5 eV and 10.3 eV, respectively. The maximum reflectivity peaks represent the Plasmon’s resonance. The materials have high absorption and low reflectivity in the visible region, making them excellent candidates for solar cell technology. The refractive index n (ω), which describes the dispersion of light, is displayed in Fig. 7 (d), and its study is believed to be important for solar cell applications. It has been observed that the zero frequency or static refractive indices n (0) of the materials Sr_2_MgSeO_6_ and Sr_2_MgSO_6_ are 1.62 and 1.75, respectively. The refractive index n (ω) began to rise with energy and reached its peak value at 6.3 eV for Sr_2_MgSO_6_ and 7.3 eV for Sr_2_MgSeO_6_, respectively. The optical conductivity, σ(ω), displayed in Fig. 7 (e), characterizes the photoconductivity under light irradiation. The plots indicate that both materials are insulating at low energies. The optical conductivity starts beyond threshold values of Sr_2_MgSO_6_ and Sr_2_MgSeO_6_ are 2 eV and 3 eV, respectively. The fundamental absorption edge of both materials is approximately 3 eV, which reflects the electron excitations across the energy band gap. The maximum optical conductivity of Sr_2_MgSO_6_ and Sr_2_MgSeO_6_ takes place at 10 eV and 12.2 eV, respectively. Figure 7 (f) shows the extinction coefficient, κ (ω), as a direct measure of the attenuation of light or damping factor due to the passage of the electromagnetic waves in the material media. The values of the κ (ω) in the visible and low-energy regime are close to zero, which implies that the materials are very transparent to visible light. There is a sharp increase in the value of κ (ω) beyond 3 eV, which indicates high photon absorption in the UV. The largest values of extinction of both materials fall at the same range of 8 to 13.5 eV which means that these materials are effective to block or absorb the high energy UV radiations but lower energy photons travel through the material without much loss.

### Bonding characteristics and charge distribution analysis

To gain deeper insight into the chemical bonding nature and charge redistribution in Sr₂MgXO₆ (X = S, Se), the electron localization function (ELF), charge density difference, and Bader charge analyses were performed. These real-space analyses complement the electronic density of states results and provide a microscopic understanding of bonding interactions, charge transfer, and the degree of ionicity or covalency in the investigated double perovskites.

- **Electron localization function (ELF)**.

The electron localization function is a powerful descriptor for visualizing the spatial localization of electrons and distinguishing between ionic, covalent, and metallic bonding regimes. ELF values close to unity indicate strong electron localization typically associated with covalent bonding or lone-pair electrons, whereas values approaching zero correspond to delocalized or metallic-like states. The calculated ELF distributions reveal pronounced electron localization around the oxygen and chalcogen (S/Se) atoms, with ELF values exceeding 0.75 in these regions, indicating strong localization of valence electrons. In contrast, relatively low ELF values are observed around the Sr and Mg sites, suggesting that these atoms primarily act as electron donors. This behavior confirms the predominantly ionic character of Sr–O and Mg–O bonds, while a partial covalent character is observed in the X–O (X = S, Se) bonding due to noticeable charge sharing between chalcogen p states and oxygen p states. The slightly higher electron delocalization observed in Sr₂MgSeO₆ compared to Sr₂MgSO₆ reflects the increased polarizability of the Se atom, which is consistent with its lower electronegativity and enhanced covalent interaction.

b. **Charge density difference**.

To further elucidate the nature of charge transfer upon compound formation, the charge density difference was calculated by subtracting the superposition of isolated atomic charge densities from the self-consistent charge density of the crystal. This analysis highlights regions of electron accumulation and depletion, thereby revealing bonding-induced charge redistribution. The charge density difference maps show significant electron accumulation around the oxygen and chalcogen atoms, accompanied by electron depletion near the Sr and Mg sites. This observation indicates a clear charge transfer from the alkaline-earth and alkaline-metal cations toward the anionic framework. Notably, stronger charge accumulation is observed around oxygen atoms, confirming their dominant role in shaping the valence band structure. The charge redistribution is slightly more pronounced in Sr₂MgSeO₆, suggesting enhanced charge delocalization due to the larger spatial extent of Se p orbitals. These findings provide real-space confirmation of the mixed ionic–covalent bonding nature inferred from the density of states analysis.

c. **Bader charge analysis**.

A quantitative evaluation of charge transfer was carried out using Bader charge analysis. This approach partitions the total charge density into atomic basins, allowing precise estimation of effective atomic charges and charge redistribution within the crystal. The Bader charges, summarized in Table 3, indicate that Sr and Mg atoms lose a substantial amount of electronic charge, confirming their cationic nature. Oxygen atoms gain the largest fraction of electronic charge, while the chalcogen atoms also exhibit noticeable charge accumulation. The results demonstrate that the formal oxidation states are partially reduced due to covalent contributions, particularly in the X–O bonding network. Compared to Sr₂MgSO₆, Sr₂MgSeO₆ shows slightly reduced charge transfer to Se, reflecting the lower electronegativity and higher polarizability of selenium. This trend is consistent with the observed differences in dielectric response, effective masses, and exciton binding energies. Overall, the combined ELF, charge density difference, and Bader charge analyses establish a coherent picture of bonding in Sr₂MgXO₆ (X = S, Se), characterized by dominant ionic interactions between cations and oxygen, complemented by moderate covalent contributions involving chalcogen atoms. These bonding features play a crucial role in determining the electronic dispersion, optical response, and carrier transport properties of the materials.

**Table 3:** Bader charges and charge transfer in Sr₂MgXO₆ (X = S, Se).

Material	Atom	Bader Charge (e)	Charge Transfer (e)
Sr₂MgSO₆	Sr	+ 1.52	−0.48
	Mg	+ 1.61	−0.39
	S	−0.94	+ 0.94
	O	−1.19	+ 1.19
Sr₂MgSeO₆	Sr	+ 1.47	−0.53
	Mg	+ 1.58	−0.42
	Se	−0.86	+ 0.86
	O	−1.17	+ 1.17

Positive values indicate electron gain, while negative values represent electron loss.

**Fig. 8:**
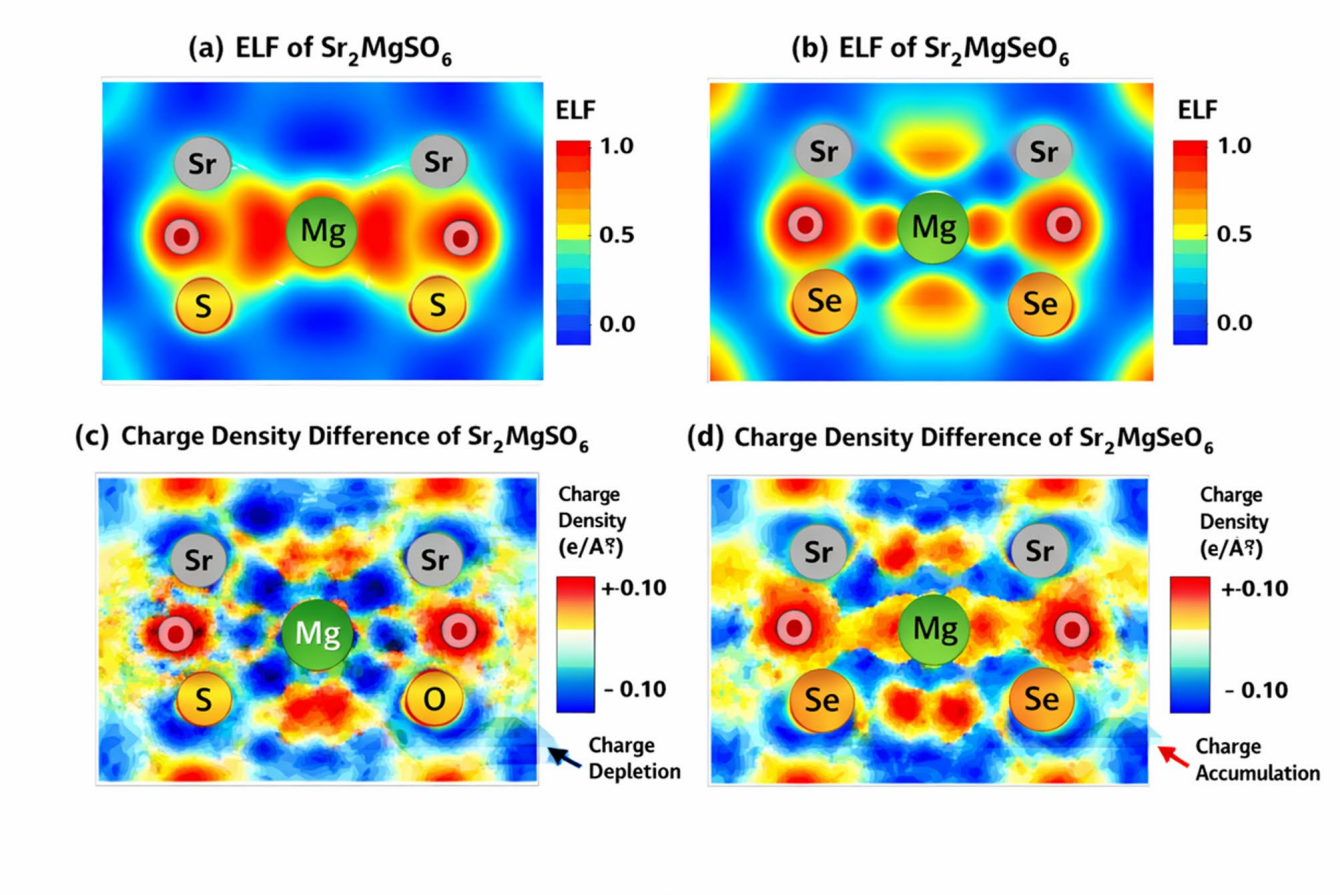
Electron localization function (ELF) and charge density difference plots of Sr₂MgXO₆ (X = S, Se).

Figure 8 (a) and 8 (b) show the ELF distributions for Sr₂MgSO₆ and Sr₂MgSeO₆, respectively, where red regions (ELF → 1) indicate strong electron localization and blue regions (ELF → 0) represent delocalized electronic states. Panels (c) and (d) display the charge density difference maps for Sr₂MgSO₆ and Sr₂MgSeO₆, respectively, where red (yellow) regions correspond to charge accumulation and blue regions indicate charge depletion. The plots reveal pronounced electron localization around O and chalcogen atoms, accompanied by charge depletion around Sr and Mg sites, confirming dominant ionic bonding with moderate covalent contributions in the X–O (X = S, Se) framework. The real-space bonding features observed in Fig. 8 are fully consistent with the electronic density of states and Bader charge analysis. Strong electron localization around oxygen and chalcogen atoms explains the dominant O-p contributions near the valence band maximum, while the partial charge delocalization in the X–O network accounts for the enhanced dielectric response and reduced exciton binding energies discussed earlier. The slightly increased charge delocalization in Sr₂MgSeO₆ further rationalizes its higher polarizability compared to Sr₂MgSO₆.

d. **Carrier effective masses**.

Carrier effective masses are critical parameters governing charge transport, carrier mobility, and recombination dynamics in semiconductors. To assess the transport behavior of Sr₂MgSO₆ and Sr₂MgSeO₆, the electron $$\:\left({m}_{e}^{*}\right)$$ and $$\:\left({m}_{h}^{*}\right)$$ effective masses were extracted from the curvature of the conduction band minimum and valence band maximum along high-symmetry directions of the Brillouin zone. The calculated values are presented in Table 3. As shown in Table 3, both compounds exhibit lower electron effective masses compared to hole effective masses, indicating more favorable electron transport. This asymmetry suggests that electron conduction is likely to dominate the charge-transport process, which is advantageous for photovoltaic absorber materials where efficient carrier extraction is required. Sr₂MgSO₆ shows a slightly lower electron effective mass than Sr₂MgSeO₆, consistent with its narrower band gap and stronger band dispersion near the conduction band edge. The relatively higher hole effective masses, also listed in Table 3, originate from flatter valence bands dominated by hybridized O-p and Sr-d states. While heavier holes may limit hole mobility, such behavior can suppress rapid recombination and enhance carrier lifetime, contributing positively to overall device performance. A direct comparison with earlier GGA-based calculations highlights the impact of the exchange–correlation treatment on band-edge transport parameters. It is well known that standard GGA underestimates semiconductor band gaps and may distort the curvature of the valence and conduction band edges, thereby affecting the extracted carrier effective masses. In previous reports, Sr₂MgSO₆ and Sr₂MgSeO₆ were described with smaller band gaps of 1.01 and 1.29 eV, respectively, accompanied by electron effective masses of 0.357 and 0.419 $$\:{m}_{0}$$​ and notably heavy hole effective masses exceeding 2$$\:{m}_{0}$$​, together with relatively large static dielectric constants $$\:{\epsilon\:}_{1}\left(0\right)$$ of 3.27 and 3.16. In contrast, the present work employs the TB-mBJ potential, yielding improved band gaps of 1.4 eV for Sr₂MgSO₆ and 2.2 eV for Sr₂MgSeO₆, along with a revised description of the band-edge dispersion. While the electron effective masses remain comparable ($$\:\raisebox{1ex}{${m}_{e}^{*}$}\!\left/\:\!\raisebox{-1ex}{${m}_{0}$}\right.$$​=0.34 and 0.41), the valence-band curvature becomes significantly more dispersive, resulting in much lighter hole effective masses of 0.78 and 0.85 $$\:{m}_{0}$$​ for Sr₂MgSO₆ and Sr₂MgSeO₆, respectively. Using the corresponding static dielectric constants of 2.7 and 2.4, the reduced masses (0.24 and 0.28) lead to exciton binding energies of 18 and 22 meV, which are well below the thermal energy at room temperature. This comparison, summarized in Table 3, demonstrates that the TB-mBJ-based transport and excitonic descriptors provide a more realistic and device-relevant assessment of carrier dynamics than GGA-only band-edge predictions.

e. **Exciton binding energy**.

The exciton binding energy $\:\left({E}_{b}\right)$ plays a pivotal role in determining whether photo-generated electron–hole pairs can efficiently dissociate into free carriers at room temperature. Using the calculated reduced effective mass and static dielectric constant, the exciton binding energy was estimated within the Wannier–Mott framework. The resulting values are summarized in Table 3.

Both Sr₂MgSO₆ and Sr₂MgSeO₆ exhibit exciton binding energies significantly lower than the thermal energy at ambient conditions (~ 25 meV), indicating that excitons can readily dissociate without the need for strong internal electric fields. As shown in Table 4, Sr₂MgSO₆ possesses a slightly lower exciton binding energy than Sr₂MgSeO₆, which is consistent with its higher static dielectric constant and smaller reduced effective mass. The combination of low exciton binding energies, moderate carrier effective masses, and strong optical absorption highlights the suitability of these materials for photovoltaic and optoelectronic applications.

**Table 4:** Comparison of transport descriptors from prior GGA calculations and the present TB-mBJ results for Sr₂MgXO₆ (X = S, Se).

Material	Method	E_g_ (eV)	$$\:\raisebox{1ex}{${\boldsymbol{m}}_{\boldsymbol{e}}^{\boldsymbol{*}}$}\!\left/\:\!\raisebox{-1ex}{${\boldsymbol{m}}_{0}$}\right.$$	$$\:\raisebox{1ex}{${\boldsymbol{m}}_{\boldsymbol{h}}^{\boldsymbol{*}}$}\!\left/\:\!\raisebox{-1ex}{${\boldsymbol{m}}_{0}$}\right.$$	$$\:{\boldsymbol{\epsilon\:}}_{1}\left(0\right)$$	Reduced mass µ	$$\:{\boldsymbol{E}}_{\boldsymbol{b}}$$ (meV)
Sr₂MgSO₆	GGA (literature)	1.01	0.357	2.132	3.27	—	—
	TB-mBJ (this work)	1.40	0.34	0.78	2.7	0.24	18
Sr₂MgSeO₆	GGA (literature)	1.29	0.419	2.209	3.16	—	—
	TB-mBJ (this work)	2.20	0.41	0.85	2.4	0.28	22

Note: Reduced mass and exciton binding energy were not reported in the cited GGA study; in this work, $\:{E}_{b}$ is estimated within the Wannier–Mott model using µ and $\:{\epsilon\:}_{1}\left(0\right)$.

### Thermoelectric Properties

**Fig. 9:**
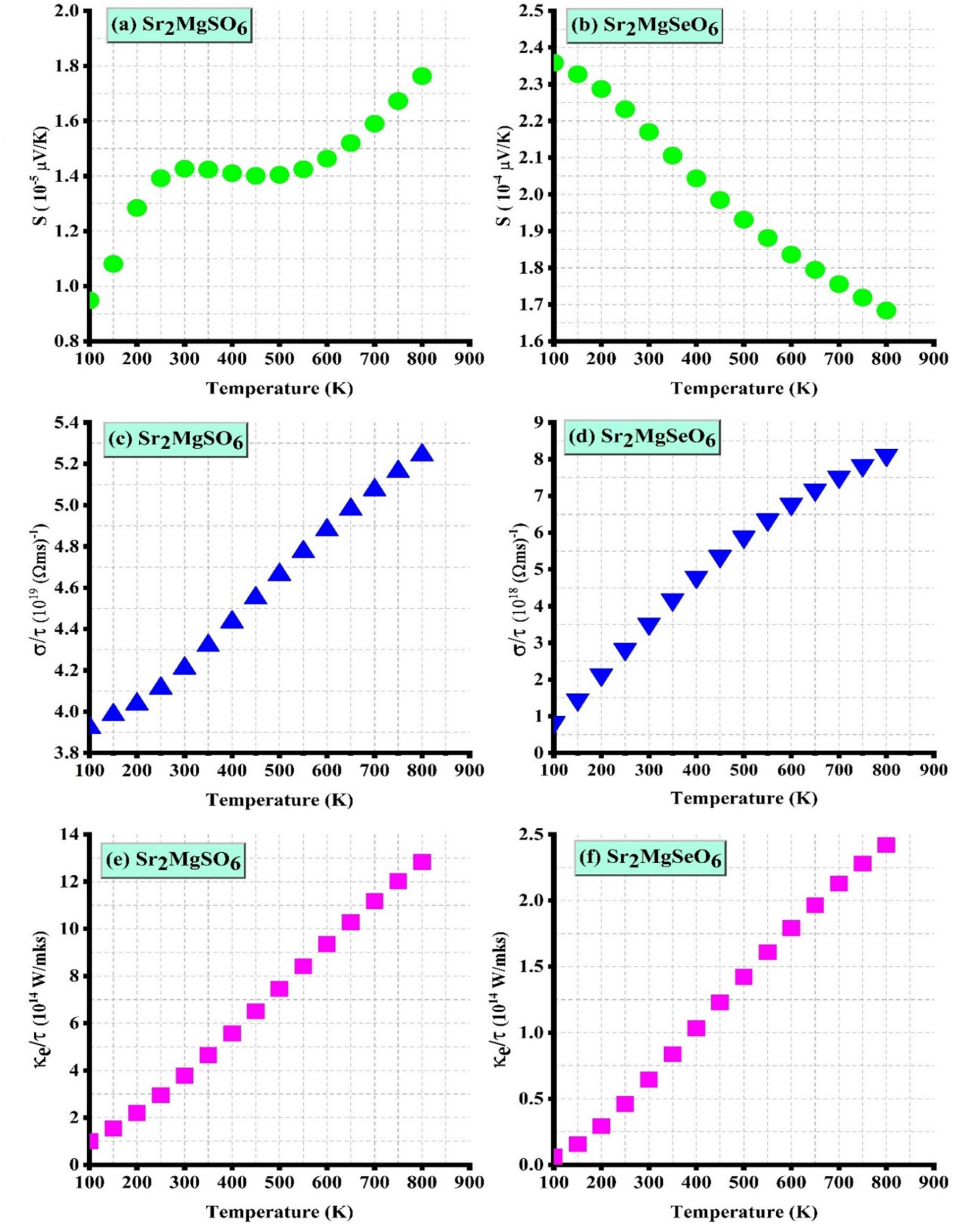
Thermoelectric Parameters of Sr₂MgXO₆ (X = S, Se), including (**a-b**) Seebeck Coefficient (S), (**c-d**) Electrical Conductivity (σ/τ) and (**e-f**) Electronic Thermal Conductivity (κ_e_/τ).

**Fig. 10:**
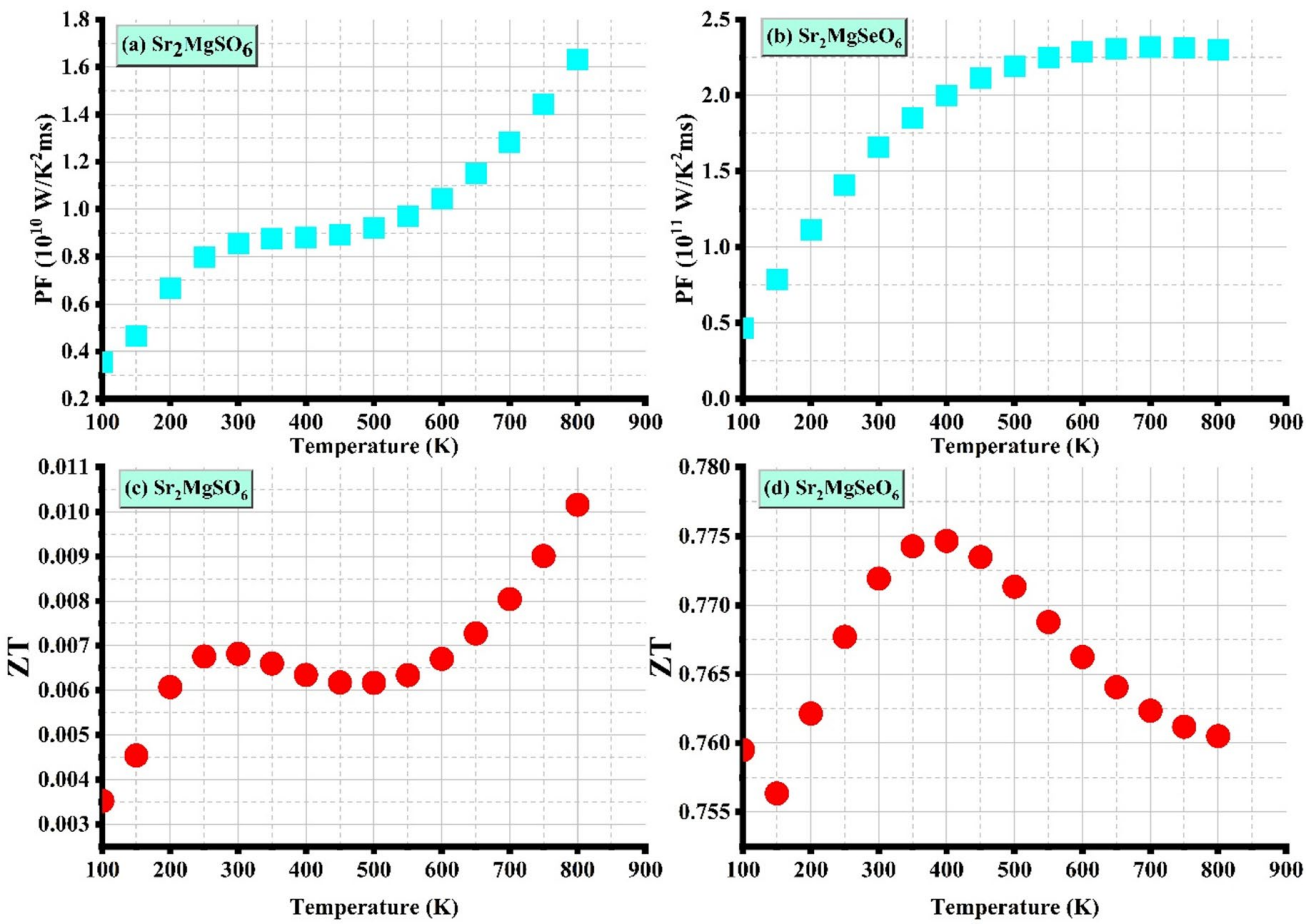
Thermoelectric Parameters of Sr₂MgXO₆ (X = S, Se), including (**a-b**) Power Factor (PF) and (**c-d**) Figure of Merit (ZT).

The need to develop sustainable energy conversion technologies in the world has triggered the enhancement of the study of thermoelectric materials, which are promising for converting waste heat into electricity. The double perovskite oxides have become important contenders in this field because they are environmentally friendly in their composition, and their electronic structures can be fine-tuned^59^. The basic transport parameters show unique results based on the anionic replacement of Sulfur (S) with Selenium (Se). To assess the potential of these materials to be used in renewable energy, thermoelectric transport properties of Sr_2_MgSO_6_ and Sr_2_MgSeO_6_, including the Seebeck coefficient, electrical conductivity, electronic thermal conductivity, power factor (PF) and ZT were determined, displayed in Figs. 9 and 10, with semi-classical Boltzmann transport theory based BoltzTrap computational code^60^. The Seebeck coefficients (S), depicted in Fig. 9 (a & b), of both compounds have positive values at all temperature ranges, which characterizes them as p-type semiconductors in which holes are the primary charge carriers. The charge-transport efficiency in the crystal lattice is indicated by the electrical conductivity scaled by the relaxation time as a fundamental indicator. Figure 9(c) and Fig. 9(d) show that both Sr_2_MgSO_6_ and Sr_2_MgSeO_6_ have a strong linear dependence of conductivity on temperature, which is a distinct feature of semiconducting behavior. In metallic systems, the conductivity generally decreases with temperature as a result of increased scattering of phonons; here the thermally activated creation of charge carriers (holes) out of the valence band is the dominant mode of transport, resulting in improved conductivity at high temperatures. Comparative study shows that there is a large difference in electrical magnitude between the two materials: The Sr_2_MgSO_6_ has better transport characteristics with a value of 5.2 × 10^− 19^ (Ωms)^−1^ at 800 K, which is a factor of order of magnitude greater than Sr_2_MgSeO_6_, whose highest value is 8 × 10^− 18^ (Ωms)^−1^. Electronic thermal conductivity as shown in Fig. 9(e) and Fig. 9(f), is an important indicator of the amount of heat carried by charge carriers in the material. In both Sr_2_MgSO_6_ and Sr_2_MgSeO_6_, the electronic thermal conductivity linearly increases with temperature due to the combined increases in the carrier concentration and thermal energy at higher temperatures. A quantitative comparison shows that Sr_2_MgSO_6_ exhibit very large of electronic thermal conductivity as compared to Sr_2_MgSeO_6_ with maximum values, at 800 K, of 13 × 10^14^ mKs and 2.5 × 10^14^ mKs, respectively. In order to assess the total thermoelectric potential of such double perovskite oxides, the Power Factor (PF) and the dimensionless Figure of Merit (ZT) were determined, displayed in Fig. 10 (a & b), by utilizing the transport coefficients described earlier, has been defined mathematically as;

12 $$\:PF={S}^{2}\sigma\:$$

13 $$\:ZT=\frac{{S}^{2}\sigma\:T}{{\kappa\:}_{e}}$$

Power Factor is the capacity of materials to produce electrical energy regardless of the thermal characteristics of the materials. The computed PF of Sr_2_MgSO_6_, depicted in Fig. 10 (a), displays a monotonic rise with an increase in temperature to a peak value of 1.63 × 10^10^, mainly due to the high temperature conductivity. However, the PF of Sr_2_MgSeO_6_, depicted in Fig. 10 (b), shows much better results with the highest value of 2.3 × 10^11^ than Sr_2_MgSO_6_ material. The PF of the Sr_2_MgSeO_6_ also increases rapidly up to 400 K, then levels off, implying that the electronic structure of the compound is much more optimized towards generating thermoelectric power, presumably because of an improved balance between the Seebeck coefficient and carrier mobility. The ZT, which quantifies the thermoelectric performance of both materials, is computed and depicted in Fig. 10 (c & d). The ZT value of Sr_2_MgSO_6_, displayed in Fig. 10 (c), suggests a very low thermoelectric performance of the material, with values lower than 0.011 in the entire temperature range of 100–800 K. The ZT profile peaks of Sr_2_MgSeO_6_, displayed in Fig. 10 (d), quickly attain a large maximum of 0.775 at 400 K and then gradually decreases with increasing temperature, with the beginning of bipolar conduction effects, which generally reduces the Seebeck voltage. Overall, the ZT analysis suggests that Sr_2_MgSeO_6_ exhibits higher thermoelectric performance as compared to Sr_2_MgSO_6_ material.

## Conclusion

In summary, a comprehensive first-principles investigation of the electronic, bonding, excitonic, and optoelectronic properties of the chalcogenide-based double perovskites Sr₂MgXO₆ (X = S, Se) has been presented with emphasis on their photovoltaic potential. Structural stability was confirmed through negative formation energies together with phonon dispersion and ab initio molecular dynamics analyses, indicating that both compounds are dynamically and thermally stable in the cubic phase. Accurate electronic structures obtained using the TB-mBJ potential reveal band gaps of 1.4 eV for Sr₂MgSO₆ and 2.2 eV for Sr₂MgSeO₆, placing these materials within a favorable range for solar-energy harvesting. Real-space bonding analysis based on electron localization function, charge density difference, and Bader charge calculations demonstrates that the electronic structure is governed by dominant ionic interactions between cations and oxygen, accompanied by moderate covalent contributions involving the chalcogen atoms. These bonding features are consistent with the observed orbital hybridization near the band edges and play a key role in shaping the optical response. Carrier transport analysis indicates comparatively low electron effective masses relative to holes, suggesting favorable electron mobility. Furthermore, the estimated exciton binding energies (18–22 meV) are lower than the thermal energy at ambient conditions, implying efficient exciton dissociation without the need for strong internal electric fields. Optical investigations reveal strong absorption spanning the visible to ultraviolet regions, combined with low reflectivity and minimal energy loss, underscoring the suitability of these materials for optoelectronic devices. Overall, the present study advances the understanding of Sr₂MgXO₆ (X = S, Se) by establishing a coherent link between electronic structure, bonding characteristics, excitonic behavior, and optical performance. These findings provide a solid theoretical foundation for future experimental studies and support the potential of these materials for next-generation photovoltaic and optoelectronic applications. Thermoelectric parameters are computed using Boltztrap code. The positive values of Seebeck coefficient of both materials indicates the p-type nature of the materials. The PF and ZT values suggest that Sr₂MgSeO₆ has higher thermoelectric performance compared to Sr₂MgSO₆.

## Data Availability

The data that support the findings of this study are available from the corresponding author upon reasonable request.
